# Associations between potentially functional *CORIN* SNPs and serum corin levels in the Chinese Han population

**DOI:** 10.1186/s12863-019-0802-4

**Published:** 2019-12-19

**Authors:** Huan Zhang, Xingbo Mo, Qiyu Qian, Zhengyuan Zhou, Zhengbao Zhu, Xinfeng HuangFu, Tan Xu, Aili Wang, Zhirong Guo, Shufeng Lei, Yonghong Zhang

**Affiliations:** 1 Jiangsu Key Laboratory of Preventive and Translational Medicine for Geriatric Diseases, Medical College of Soochow University, Suzhou, Jiangsu People’s Republic of China; 2 Department of Epidemiology, School of Public Health, Medical College of Soochow University, Suzhou, 199 Renai Road, Industrial Park District, Suzhou, 215123 Jiangsu People’s Republic of China; 3 Center for Genetic Epidemiology and Genomics, School of Public Health, Medical College of Soochow University, Suzhou, Jiangsu People’s Republic of China; 4 Changshu Center of Disease Control and Prevention, Suzhou, Jiangsu People’s Republic of China

**Keywords:** Corin, Natriuretic peptide, Hypertension, Methylation, Mendelian randomization

## Abstract

**Background:**

Corin is an important convertase involved in the natriuretic peptide system and may indirectly regulate blood pressure. Genetic factors relate to corin remain unclear. The purpose of the current study was to comprehensively examine the associations among *CORIN* SNPs, methylations, serum corin levels and hypertension.

**Results:**

We genotyped 9 tag-SNPs in the *CORIN* gene and measured serum corin levels in 731 new-onset hypertensive cases and 731 age- and sex-matched controls. DNA methylations were tested in 43 individuals. Mendelian randomization was used to investigate the causal associations. Under additive models, we observed associations of rs2289433 (p.Cys13Tyr), rs6823184, rs10517195, rs2271037 and rs12509275 with serum corin levels after adjustment for covariates (*P* = 0.0399, 0.0238, 0.0016, 0.0148 and 0.0038, respectively). The tag-SNP rs6823184 and SNPs that are in strong linkage disequilibrium with it, i.e., rs10049713, rs6823698 and rs1866689, were associated with *CORIN* gene expression (*P* = 2.38 × 10^− 24^, 5.94 × 10^− 27^, 6.31 × 10^− 27^ and 6.30 × 10^− 27^, respectively). Neither SNPs nor corin levels was found to be associated with hypertension. SNP rs6823184, which is located in a DNase hypersensitivity cluster, a CpG island and transcription factor binding sites, was significantly associated with cg02955940 methylation levels (*P* = 1.54 × 10^− 7^). A putative causal association between cg02955940 methylation and corin levels was detected (*P* = 0.0011).

**Conclusion:**

This study identified potentially functional *CORIN* SNPs that were associated with serum corin level in the Chinese Han population. The effect of *CORIN* SNPs on corin level may be mediated by DNA methylation.

## Background

Hypertension is identified as one of the most important causal risk factors for cardiovascular diseases, which is the leading cause of death worldwide [1, 2]. The levels of diagnosis, treatment and control of hypertension are much lower in China than in Western countries. The China Kadoorie Biobank Study, a nationwide investigation in 500,223 people, showed that the prevalence of hypertension has already increased to 32.5% during year 2004 to 2009 [3].

The natriuretic peptide system regulates salt, water balance and blood pressure (BP) [4]. Corin, a type II transmembrane serine protease found in cardiomyocytes, converts the precursor molecules of A-type natriuretic peptides (ANP) into active proteins [5]. Deficiency in corin is expected to reduce ANP production and cause hypertension [5]. Variants in the human *CORIN* gene have been identified to alter corin protein conformation and inhibited corin zymogen activation and contribute to hypertension and cardiovascular diseases [6, 7].

The prospective association of serum corin level with hypertension has not been assessed in a Chinese Han population. On the other hand, genetic and epigenetic factors that affect serum corin level have not been identified. What’s more, although previous genetic association studies examined the associations of *CORIN* SNPs with hypertension [8, 9], few studies have tried to explore the functional variants to explain the associations and mechanisms [10]. Therefore, we conducted this study to (1) evaluate the effect of serum corin level on hypertension, (2) evaluate the effects of SNPs in *CORIN* gene on serum corin level and (3) hypertension, and (4) find out the potentially functional variants. Firstly, we examined the association between serum corin level and new-onset hypertension in a nested case-control study from a large prospective cohort of non-hypertensive people who were followed-up over a 6-year period. Then we tested whether SNPs in *CORIN* gene were associated with baseline serum corin level and hypertension in the same sample. In addition, we evaluated the functionalities of the associated SNPs and SNPs in strong linkage disequilibrium (LD) with them by bioinformatics analysis. Finally, we applied Mendelian randomization to identify putative causal factors, e.g., DNA methylation.

## Methods

### Study population

The study was a nested case-control study from a large prospective cohort of non-hypertensive people in Jiangsu Province, China. The baseline survey among 12,423 participants has been completed in 2007–2008. Participants with cardiovascular diseases, diabetes mellitus, nephropathy, hypertension, or were taking antihypertensive medication were excluded at baseline. The follow-up study has been completed in 2013, and a total of 1774 participants were found to be hypertensive [11]. Among the 1774 patients with new-onset hypertension, we selected 740 new-onset hypertension patients who have both serum and DNA samples as cases by means of stratified random sampling method according to their resident areas. We selected half of the 1774 incident hypertensive individuals because the high cost of the testing of serum corin levels and genotyping. The paired controls were chosen in the follow-up study. Written informed consent was obtained from each of the study participants. This study was approved by the ethics committee at Soochow University in China.

### Baseline examination

The survey methods have been described previously [12]. In brief, basic information for all participants was obtained using a standard questionnaire administered by trained staff. Three sitting BP measurements were taken at 30-s intervals by trained observers using a standard mercury sphygmomanometer after the subjects had been resting for 5 min according to a standard protocol. The first and fifth Korotkoff sounds were recorded as the systolic (SBP) and diastolic (DBP) blood pressures, respectively. The mean of the three BP measurements was used in the analysis. Fasting blood samples were collected for laboratory tests, including total cholesterol (TC), triglycerides (TG), high-density lipoprotein cholesterol (HDL-C), and fasting blood glucose (FBG). Low-density lipoprotein cholesterol (LDL-C) levels were calculated using the Friedewald equation for participants who had less than 400 mg/dL TG.

### Follow-up study

All participants were followed up in 2013. The information of demographic characteristics, lifestyle risk factors, personal medical history and family history of hypertension for all participants was also obtained using a standard questionnaire administered by trained staff. The questionnaires and measuring methods used in the follow-up study were the same as those used at baseline. Three sitting BP measurements were taken at 30-s intervals by trained observers using a standard mercury sphygmomanometer after the subjects had been resting for 5 min according to a standard protocol. The first and fifth Korotkoff sounds were recorded as the SBP and DBP, respectively. The mean of the three BP measurements was used in the analysis. Hypertension was defined as SBP ≥ 140 mmHg and/or DBP ≥ 90 mmHg or use of antihypertensive medication in the last 2 weeks [13]. Sex- and age-matched (±2 years) controls were selected. The controls were normotensive participants during the follow-up throughout the same time interval as the hypertensive cases.

### Measurement of corin

Blood samples were collected for all of the participants in the morning after at least 8 h of fasting. The serum samples were frozen at − 80 °C until the laboratory testing was performed. We used ELISA kits (R&D Systems, Minneapolis, MN; Catalog: DCRN00) to test the serum corin levels. All analyses were performed by the same lab and all of the samples were processed in a duplicate assay. Standard curves were constructed from which corin concentrations of unknown samples were determined. Intra- and inter-assay coefficients of variation were less than 5%.

### SNP selection and genotyping

We selected tag-SNPs for the *CORIN* gene by using the Haploview software (version 4.2, http://www.broad.mit.edu/mpg/haploview) with LD r^2^ thresholds of 0.8 [14]. A total of 9 tag-SNPs were selected and genotyped by using SNPscan™ technology, a custom-by-design 48-Plex Kit based on double ligation and multiplex fluorescence PCR (Catalog: G0104; Genesky Biotechnologies Inc., Shanghai, China). This kit was developed according to patented SNP genotyping technology by Genesky Biotechnologies Inc., which was based on double ligation and multiplex fluorescence PCR.

### Detection of potentially functional variants

We detected putative functional variants which are in strong LD (r^2^ > 0.8, based on data of 1000G Phase 1 of East Asian population) with the tag-SNPs that were detected to be associated with serum corin level or hypertension. Several kinds of functional variants were considered in this study, including missense mutations, SNPs with specific functions such as N^6^-methyladenosine (m^6^A)-associated SNPs [15] and phosphorylation-related SNPs (phosSNPs) [16], SNPs have regulatory potentials, as well as expression quantitative trait loci (eQTL) in *CORIN*. The m^6^A-associated SNPs were found in the m6AVar database (http://m6avar.renlab.org/), and the phosSNPs were found in the phosSNP 1.0 database (http://phossnp.biocuckoo.org/index.php). eQTLs and SNPs with potential functionalities in transcription regulation were found in HaploReg (https://pubs.broadinstitute.org/mammals/haploreg/haploreg.php).

### Causal inference

We examined whether the identified SNPs were associated with methylation levels by using data extracted from our in-house datasets of 43 female subjects from the Chinese Han population (GSE111942). Basic characteristics of the study subjects have been described in a previous study [17]. The study was approved by the ethical committee of Soochow University. The written informed consent was obtained from each of the participants. Affymetrix Genome-Wide Human SNP Array 6.0 chips were employed for SNP genotyping. DNA methylation profiling was performed using Illumina 450 K Infinium Methylation BeadChip (Illumina, Inc., USA) according to the manufacturer’s instructions. The associations between SNPs and methylations were detected by using the R package MatrixeQTL [18]. Finally, we applied the gsmr R-package which implements the Generalised Summary-data-based Mendelian Randomization (GSMR) method [15, 19] to test for putative causal association between methylation and corin levels using summary-level data on the associations of SNPs with methylations and associations of the same SNPs with corin levels. Compare with other Mendelian randomization approaches, the GSMR method doesn′t need individual-level raw data that was tested in the same samples, and it can work without data on the associations between methylation levels (risk factor) and corin levels (outcome). This method facilitates the integration of the methylation data and the corin level data from independent studies via the instrument variables. The tested SNPs that were significantly associated with methylation levels (Bonferroni correction *P* < 5.56 × 10^− 3^) were used as instrument variables, because a strong association between instrument variable and exposure is needed [20–22]. The genotype data of the 9 tested *CORIN* SNPs in 1472 individuals were used to calculate the LD correlation to construct the LD correlation matrix for GSMR analysis. The association threshold to select SNPs as the instruments in the GSMR analysis was set to be 5.56 × 10^− 3^ and the HEIDI-outlier threshold was 0.05. For other parameters the default setting was used.

### Statistical analysis

The differences of baseline characteristics between hypertensive cases and controls were compared, using Student’s *t*-test for continuous variables and χ^2^ tests for categorical variables. The study participants were categorized into 4 groups according to the quartiles of serum corin levels. Odds ratios (ORs) and 95% confidence intervals (CIs) of hypertension were calculated for upper quartiles of corin levels with the lowest quartile as references by using conditional logistic regression models. Potential covariates were smoking, drinking, BMI, TC, TG, HDL-C, FBG, and family history of hypertension. Hardy–Weinberg equilibrium (HWE) of the selected SNPs was tested with Fisher’s exact test among control participants. Conditional logistic regression models were used to calculate ORs and 95%CIs of hypertension with SNPs. SNP was analyzed as 0, 1 or 2 copies of the minor allele in an additive genetic model. Kruskal-Wallis test was applied to test the associations between the SNPs and baseline serum corin levels, and methylation levels. Statistical analyses were conducted using SAS statistical software version 9.2 (SAS Institute, Cary, NC).

## Results

### Baseline characteristics

In all, 731 hypertensive cases and 731 age- and sex-matched controls with full data of SNP genotypes, serum corin measurements were included in the analyses. Table 1 shows the baseline characteristics of the study cohort. There were 283 (38.45%) men in each group. The mean age at baseline was 52.85 years old in hypertensive participants and 52.73 in controls. Compared with the controls, mean baseline BMI, SBP, DBP, TC, TG and FBG levels, follow-up SBP and DBP, and frequencies of drinkers were all higher in hypertensive patients (*P* < 0.05) (Table 1). The mean SBP is 146.8 mmHg and mean DBP is 82.6 mmHg in cases. It may be due to two reasons: 1) 62 individuals with DBP ≥ 90 mmHg but SBP < 140 mmHg and 437 individuals with SBP ≥ 140 mmHg but DBP < 90 mmHg; 2) 239 patients took hypotensive drugs.

**Table 1:** Characteristics of study participants

Characteristics^a^	Hypertensives (*n* = 731)	Controls (*n* = 731)	*P* value
Age, year	52.85 ± 12.79	52.73 ± 12.62	0.8579
Male, %	38.45	38.45	1.000
Smokers, %	28.45	28.86	0.8622
Drinkers, %	23.94	19.02	0.0219
Family history of hypertension, %	38.44	34.75	0.1427
Baseline SBP, mmHg	126.4 ± 8.37	118.2 ± 10.50	< 0.0001
Baseline DBP, mmHg	78.27 ± 6.81	72.93 ± 7.25	< 0.0001
Follow-up SBP, mmHg	146.8 ± 11.56	121.1 ± 10.54	< 0.0001
Follow-up DBP, mmHg	82.6 ± 10.26	70.67 ± 7.99	< 0.0001
BMI, kg/m^2^	22.89 ± 3.27	21.57 ± 2.74	< 0.0001
TC, mmol/L	4.58 ± 1.04	4.47 ± 0.91	0.0366
TG, mmol/L	1.58 ± 1.13	1.35 ± 0.86	< 0.0001
HDL-C, mmol/L	1.30 ± 0.47	1.34 ± 0.48	0.0972
LDL-C, mmol/L	2.53 ± 0.85	2.46 ± 0.74	0.1245
FBG, mmol/L	5.12 ± 1.08	5.01 ± 0.81	0.0306
Corin, pg/mL	578.4 ± 271.0	585.4 ± 258.5	0.6157

^a^: All variables presented are collected at baseline, except follow-up blood pressure

### Association between genotypes and corin levels and hypertension

HWE of the 9 SNPs was tested with Fisher’s exact test and no departure was observed (Table 2). Under additive models, we observed associations of rs2289433 (p.Cys13Tyr), rs6823184, rs10517195, rs2271037, and rs12509275 with serum corin levels after adjustment for covariates (*P* = 0.0399, 0.0238, 0.0016, 0.0148 and 0.0038, respectively) (Table 2). However, if we considered multiple testing for the 9 *CORIN* SNPs, only the associations between rs10517195 and rs12509275 and serum corin levels were significant (*P* < 0.05/9 = 0.0056). Table 2 also showed the results of association between genotypes and hypertension. No significant association was detected in either univariate analysis or multivariate analysis.

**Table 2:** Association between *CORIN* gene polymorphisms and serum corin levels and incident hypertension

SNP	Position	MA	MAF %	HWE^*^	Corin				Hypertension			
					Beta	*P*	Beta^#^	*P* ^#^	OR(95%CI)	*P*	OR(95%CI)^#^	*P* ^#^
rs2289433	exon 1	G	34.3	0.075	0.035	0.052	0.037	0.040	1.08(0.92,1.26)	0.358	1.13(0.96,1.34)	0.152
rs6823184	intron 1	C	43.5	0.870	0.036	0.033	0.038	0.024	0.95(0.82,1.11)	0.509	0.90(0.77,1.06)	0.220
rs10008014	intron 6	C	24.6	0.289	−0.002	0.883	0.000	0.990	0.99(0.84,1.18)	0.931	1.02(0.85,1.22)	0.853
rs17654423	intron 6	C	26.6	0.670	−0.020	0.510	− 0.019	0.319	1.02(0.87,1.20)	0.801	1.02(0.86,1.22)	0.804
rs10517195	exon 9	G	15.7	0.167	0.074	0.001	0.074	0.002	0.94(0.77,1.16)	0.568	0.93(0.76,1.16)	0.515
rs2271037	intron 9	T	38.7	0.551	0.042	0.022	0.042	0.015	1.01(0.87,1.17)	0.911	0.97(0.83,1.14)	0.736
rs2351784	intron 11	T	23.0	0.335	−0.014	0.615	−0.012	0.854	0.92(0.77,1.10)	0.348	0.90(0.75,1.09)	0.288
rs12509275	intron 17	G	13.8	0.064	0.073	0.003	0.070	0.004	1.01(0.82,1.25)	0.913	0.94(0.75,1.18)	0.573
rs3749585	exon 22	C	48.0	0.774	−0.001	0.814	−0.006	0.737	0.99(0.85,1.14)	0.852	0.98(0.84,1.15)	0.834

*HWE* Hardy–Weinberg equilibrium; *MA* Minor allele; *MAF* Minor allele frequency

*: *P* value for HWE using Fisher’s exact test

**#**: Adjusted for smoking, drinking, BMI, TC, TG, HDL-C, FBG, family history of hypertension

**Fig. 1:**
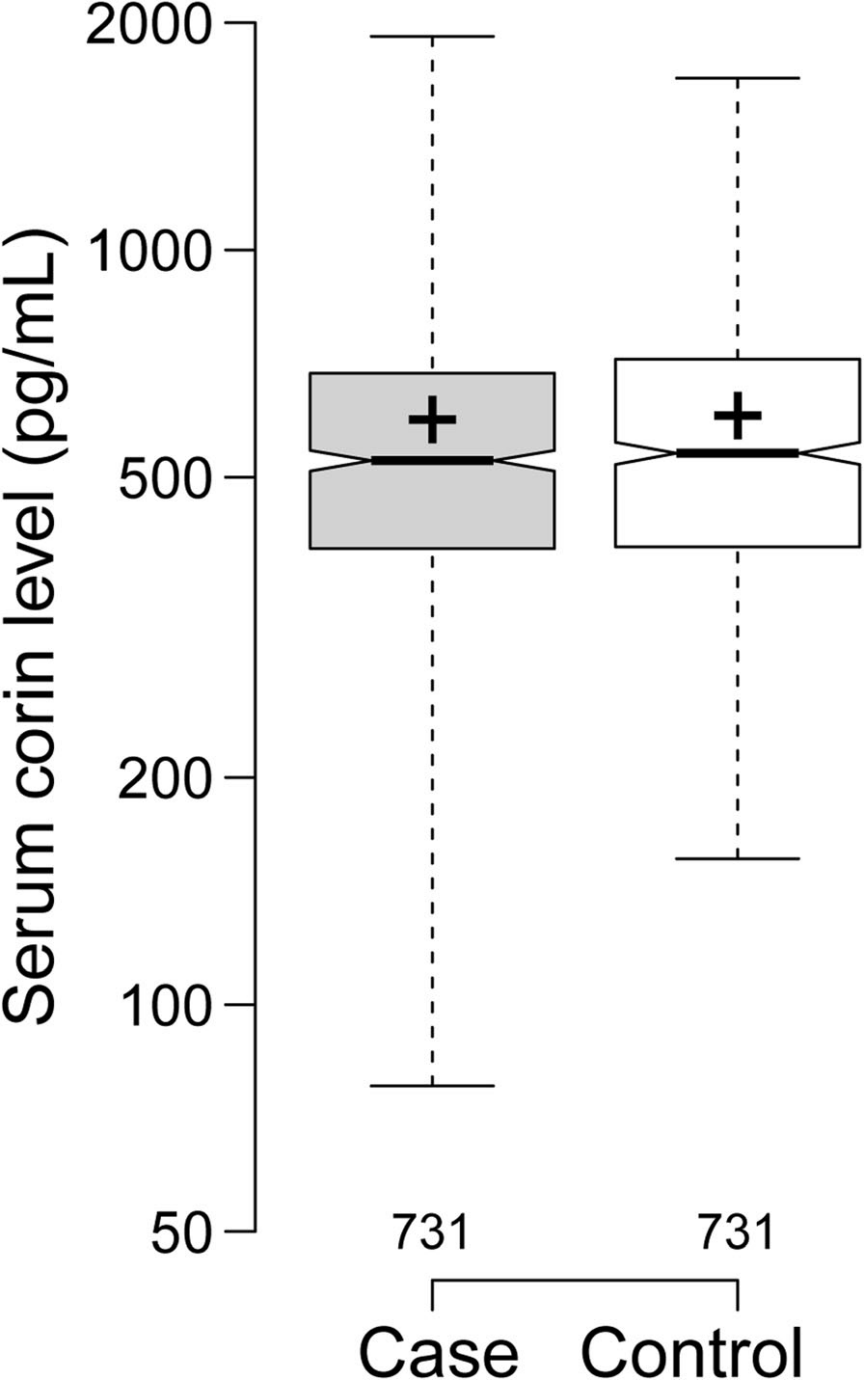
Serum corin levels between hypertensive cases and controls

Serum corin levels were lower in hypertensive patients (578.4 ± 271.0 pg/mL) than in controls (585.4 ± 258.5 pg/mL) but not significant (Fig. 1). Conditional logistic regression models were used to assess the association of serum corin levels with hypertension. In univariate and multivariate analysis, the association between serum corin levels and hypertension was not significant (Additional file 1: Table S1).

### Detection of functional variants

**Fig. 2:**
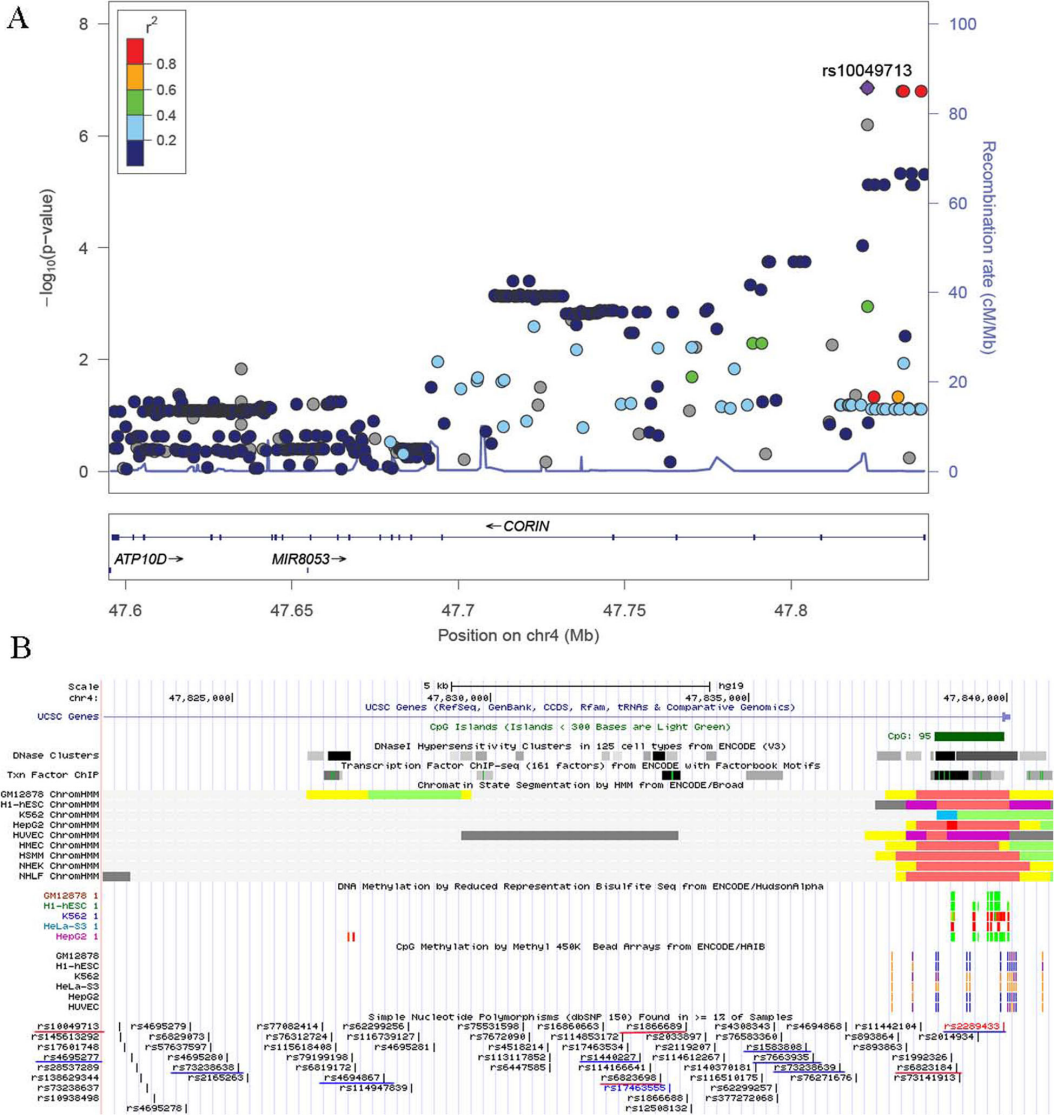
Relationships between *CORIN* SNPs and DNA methylation. **a** Regional plot for the associations between *CORIN* SNPs and cg02955940 methylation level. **b** The genomic region of intron 1 of *CORIN* gene. The 9 tracks sequentially show base position, UCSC gene, CpG island, DNaseI hypersensitivity clusters, transcription factor, chromatin state segmentation by HMM, DNA methylation, CpG methylation from ENCODE, and common SNPs. The 12 underlined SNPs were very strongly associated with cg02955940 methylation levels (Additional file 3: Table S3)

The genetic association tests found 5 tag-SNPs that were associated with serum corin level. Therefore, we searched for functional variants which are in strong LD with the 5 corin-associated tag-SNPs, i.e., rs2289433, rs6823184, rs10517195, rs2271037 and rs12509275. There were 132 SNPs that are in strong LD with the 5 associated SNPs. So we focused on these 137 SNPs in the bioinformatics analysis (Additional file 2: Table S2). In HaploReg, we found that most of the SNPs overlap promoter, enhancer and DNase in multiple tissues. Several SNPs, such as tag-SNPs rs6823184, rs3215139, rs1344122, rs4695263 and rs4695264 might alter the binding of one or more proteins, and 126 SNPs might alter regulatory motifs in different cell types from the ENCODE transcription factor ChIP-seq datasets [23] (Additional file 2: Table S2). Among these SNPs, rs10049713, rs6823698, rs1866689 and the tag-SNPs rs6823184 were associated with expression of *CORIN* gene in whole blood cells (GTEx 2015_v6) (the top *P* values = 2.38 × 10^− 24^, 5.94 × 10^− 27^, 6.31 × 10^− 27^ and 6.30 × 10^− 27^, respectively). The associations in other tissue such as Esophagus Muscularis were also observed. These four SNPs locate in intron 1 of *CORIN*. We found that rs6823698 locate in a DNase hypersensitivity cluster, and rs6823184 locate in a DNase hypersensitivity cluster, a CpG island and transcription factor binding sites (Fig. 2).

### Causal inference

**Fig. 3:**
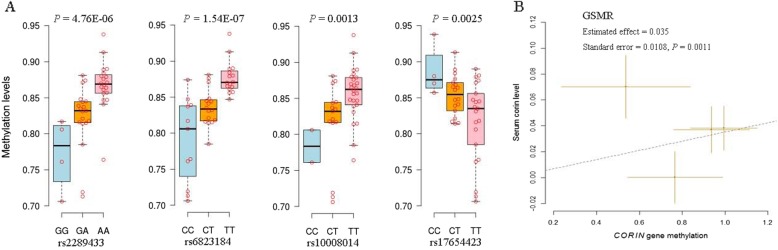
Relationship between *CORIN* SNPs, methylation and corin levels. **a** Four SNPs, including rs2289433, rs6823184, rs10008014 and rs17654423, were significantly associated with cg02955940 methylation levels. **b** We detected a putative causal association between cg02955940 methylation and corin levels by using these *CORIN* SNPs as genetic instruments in Mendelian randomization analysis (Additional file 4: Table S4)

According to the bioinformatics analysis, we noticed that the corin-associated SNPs locate around a CpG island. So we tested the associations between *CORIN* SNPs and methylation levels. Twenty-five methylation sites in *CORIN* were analyzed. We found 12 significant (FDR < 0.05) associations between 12 SNPs and cg02955940 methylation sites in *CORIN* gene (Fig. 2, Additional file 3: Table S3). The associations between the 9 tag-SNPs genotyped in our study and methylation levels were evaluated. Four of the *CORIN* tag-SNPs seemed to be associated with cg02955940 (*P* < 2.54 × 10^− 3^). For the corin-associated tag-SNPs, the association between rs6823184 and cg02955940 methylation level was the most significant (*P* = 1.54 × 10^− 7^) (Fig. 3a, Additional file 4: Table S4). We used these 9 *CORIN* SNPs that have data on both of the associations of SNPs with serum corin levels and the associations of SNPs with methylation levels as genetic instruments in Mendelian randomization analysis. Four SNPs that were significantly associated with methylation (*P* < 2.54 × 10^− 3^) were retained after filtering and passed the HEIDI-outlier test. Thus, these four SNPs were the instrumental variables in the GSMR analysis. The estimated effect of cg02955940 methylation on corin levels was 0.035 (standard error = 0.0108, *P* = 0.0011) (Fig. 3b). This result suggested a putative causal association between cg02955940 methylation and corin levels.

## Discussion

This is the first study to investigate the relationships among *CORIN* SNPs and methylations, serum corin levels and risk of hypertension in China. Our study suggested that *CORIN* SNPs were associated with serum corin levels and cg02955940 methylation was likely to be a causal factor for corin levels.

Human ANP exerts an important role in regulation of systemic BP and intravascular volume by enhancing vascular permeability [24, 25]. Low ANP level is a risk factor for hypertension [26, 27]. Deficiency in corin leads to reduced ANP production [5]. The importance of corin in regulating BP has been evaluated. In corin-null mice, SBP, DBP and mean arterial BP were increased compared to the normal controls [5]. Corin has been reported to be a valuable prognostic marker of major adverse cardiac events in patients with acute myocardial infarction, independent of established conventional risk factors [28]. A previous cross-sectional study has detected the significant higher level of serum soluble corin in hypertensive patients [7], which was inconsistent with the hypothesis from cell- and animal-based studies that increased corin level probably lowered BP [5, 6]. In the present study, we prospectively evaluated the association between baseline corin levels and incident hypertension but did not detect a significant association. However, the mean corin level of hypertension patients at baseline was lower than that of normotensive participants, which was consistent with the hypothesis. Corin is a converting enzyme in the natriuretic peptide system. Changes in activity or concentration of this convertase would affect ANP concentration and then influence hypertension risk. Even though, the function of this protein on BP regulation should not be as direct as ANP. The effect size of corin on hypertension incidence may be too small to detect.

The associations between *CORIN* gene SNPs and serum corin levels have not been comprehensively assessed in previous studies. The two well-known polymorphisms, T555I and Q568P, are most likely to be monomorphisms in the Chinese Han population (we have also genotyped these two SNPs in 1480 individuals). The effects of these two variants can’t be evaluated in our study. Taking no account of these two SNPs, we identified several SNPs that were significantly associated with serum corin level. Moreover, the effects of these SNPs on corin level may be mediated by DNA methylation. Therefore, our data suggested that genetic variants in *CORIN* gene may affect serum corin levels. To our knowledge, these associations were first reported.

In African Americans, Dries et al. had reported the associations of *CORIN* variants (T555I and Q568P) with hypertension and heart disease [29]. Later, other polymorphisms have been reported [30]. But these associations have not been confirmed in recent large scale genome-wide association studies. A recent study evaluated the associations of three *CORIN* SNPs and BP in a Chinese population but no significant association was detected [9], while another study concluded that rs2271037 and rs3749585 were significantly associated with hypertension in a Han population of northeastern China [8]. Although data from several studies suggested that *CORIN* variants may contribute to hypertension and heart disease in this high-risk population [29, 31–33], the association between *CORIN* gene variants and incident hypertension has not yet been reported. We evaluated the association between *CORIN* gene variants and incident hypertension but fail to detect a significant result. Given the fact that BP is a complex trait, which influenced by multiple interacting physiologic regulatory systems where multiple genes are likely to be involved, small genetic effects can be expected. The effects of *CORIN* gene variants on hypertension risk were indirect and would be very small.

This study has several strong points. First, we investigated the association between baseline serum corin level and the risk of incident hypertension in a nested case-control study. The prospective design reduced the potential biases inherent in cross-sectional or retrospective studies. Second, simultaneously testing several tag-SNPs and serum corin levels in a sample ensured us a comprehensive analysis of the relationships among SNPs, corin levels and hypertension incidence. Finally, and most importantly, we found potentially functional variants in the *CORIN* gene according to data from functional annotation databases and data from large projects such as ENCODE, which might consequently facilitate the interpretation of the associations. The observation that corin-associated SNPs locate in a CpG island guided us to evaluate the relationship among SNPs, methylations and corin levels, which enables us to discover the causal effect of methylation on corin level.

Although we were not able to link serum corin levels to hypertension in a Chinese Han population, potentially functional SNPs were found to be significantly associated to serum corin level, and the effect of these SNPs on corin level may be mediated by DNA methylation. These findings were first reported. Previous findings in cells, animals and the general populations supported the interest of investigations on corin, but the regulatory mechanism is far from fully understood. Our findings provided new clues for further investigation in order to unfold the mechanisms involved.

Our study has some potential limitations. First, the study sample was too small to detect the associations between *CORIN* SNPs and hypertension. We displayed power estimations by using the CaTS software. We found that no SNP has enough power (< 80%) to detect the association in an additive model. Larger samples are needed to detect the association between *CORIN* SNPs and hypertension. For example, we have 38% power to detect the association between rs16823124 and hypertension in the current samples. For this SNP, if the sample size is increased to thousands of individuals (3000 cases and 3000 controls) the statistical power may be high enough (84%). Second, we did not test the corin activities or gene expressions to evaluate the effects. However, several molecular genetic studies have shown that the *CORIN* mutations may adversely affect the biological activity of corin [30, 31, 33], and we have detected *cis*-eQTL effects for these SNPs. Third, although we detected the strong associations between *CORIN* SNPs and methylation, the sample is relatively small. The relationship among SNPs, methylations and corin levels needs to be confirmed in larger samples.

## Conclusion

In summary, this is the first study to show that potentially functional variants in *CORIN* gene were associated with serum corin level in the Chinese Han population. The effect of these SNPs on corin level may be mediated by *CORIN* methylation. Further studies are needed to confirm the associations and elucidate the mechanism.

## Supplementary information

**Additional file 1: Table S1.** Odds ratios and 95% confidence interval of hypertension associated with quartiles of Corin. The results showed that in univariate and multivariate analysis, the association between serum corin levels and hypertension was not significant.

**Additional file 2: Table S2.** Genetic variants in strong linkage disequilibrium with the corin-associated SNPs. There were 132 SNPs that are in strong LD with the 5 corin-associated SNPs. This table showed the bioinformatics analysis results for these 137 SNPs.

**Additional file 3: Table S3.** Significant associations between *CORIN* SNPs and methylations. The results showed 12 significant associations between 12 SNPs and cg02955940 methylation sites in CORIN gene (FDR < 0.05).

**Additional file 4: Table S4.** Summary association data for GSMR analysis. This table contained the formatted summary-level data that was used in the GSMR analysis.

## Data Availability

The datasets supporting the conclusions of this article are included within the article and its additional files.

## Abbreviations

ANP: A-type natriuretic peptides
BMI: Body mass index
BP: Blood pressure
CI: Confidence intervals
DBP: Diastolic blood pressure
eQTL: Expression quantitative trait loci
FBG: Fasting blood glucose
GSMR: Generalised Summary-data-based Mendelian Randomization
HDL-C: High-density lipoprotein cholesterol
LD: Linkage disequilibrium
MAF: Minor allele frequency
OR: Odds ratios
SBP: Systolic blood pressure
SNP: Single nucleotide polymorphism
TC: Total cholesterol
TG: Triglycerides
