# Supplementary material for: Associations between potentially functional CORIN SNPs and serum corin levels in the Chinese Han population
Source: BMC Genet. 2019 Dec 19;20:99. doi: 10.1186/s12863-019-0802-4 (PMC6923953; doi:10.1186/s12863-019-0802-4)
Supplement: Supplementary file 1 — Additional file 1: Table S1. Odds ratios and 95% confidence interval of hypertension associated with quartiles of Corin. The results showed that in univariate and multivariate analysis, the association between serum corin levels and hypertension was not significant. [file 12863_2019_802_MOESM1_ESM.pdf]

Supplementary Table S1 Odds ratios and 95% confidence interval of hypertension associated with quartiles of Corin

**Groups**	**Unadjusted**		**Adjusted***	
	**OR（95%CI）**	***P* value**	**OR（95%CI）**	***P* value**
＜402	1		1	
402~534	0.935（0.697, 1.252）	0.6501	0.882（0.644, 1.208）	0.4337
534~704	1.060（0.795, 1.413）	0.6936	1.087（0.801, 1.475）	0.5926
≥704	0.835（0.622, 1.120）	0.2291	0.830（0.606, 1.136）	0.245

*Adjusted for smoking, drinking, BMI, TC, TG, HDL-C, FBG, family history of hypertension.
