# Supplementary material for: Associations between potentially functional CORIN SNPs and serum corin levels in the Chinese Han population
Source: BMC Genet. 2019 Dec 19;20:99. doi: 10.1186/s12863-019-0802-4 (PMC6923953; doi:10.1186/s12863-019-0802-4)
Supplement: Supplementary file 2 — Additional file 2: Table S2. Genetic variants in strong linkage disequilibrium with the corin-associated SNPs. There were 132 SNPs that are in strong LD with the 5 corin-associated SNPs. This table showed the bioinformatics analysis results for these 137 SNPs. [file 12863_2019_802_MOESM2_ESM.pdf]

Supplementary Table S2 Genetic variants in strong linkage disequilibrium with the corin-associated SNPs

| chr | position (hg38) | LD($r^2$) | Genetic variant | Ref allele | Alt allele | freq (ASN) | Promoter histone marks | Enhancer histone marks | DNAse | Proteins bound | Motifs changed | GWAS hits | GRASP QTL hits | Selected eQTL hits | GENCODE genes | function |
|---|---|---|---|---|---|---|---|---|---|---|---|---|---|---|---|---|
| SNP rs2289433 and variants with $r^2$> 0.8 | | | | | | | | | | | | | | | | |
| 4 | 47821040 | 0.93 | rs4695277 | G | A | 0.26 | | | | | GCNF,HNF4,Pax-6 | | | | CORIN | intronic |
| 4 | 47823103 | 0.91 | rs73238638 | G | A | 0.26 | | HRT | | | | | | | CORIN | intronic |
| 4 | 47825911 | 0.93 | rs4694867 | C | T | 0.26 | | 4 tissues | HRT | | | | | | CORIN | intronic |
| 4 | 47830875 | 1 | rs1440227 | T | C | 0.72 | HRT | 6 tissues | BLD | | AP-4,LBP-1 | | 4 hits | | CORIN | intronic |
| 4 | 47832134 | 0.93 | rs2033897 | G | A | 0.26 | HRT | | | | Hmx,Hoxb7,Hoxb8 | | 1 hit | | CORIN | intronic |
| 4 | 47834079 | 0.92 | rs1583808 | C | T | 0.27 | HRT | | | | 4 altered motifs | | | | CORIN | intronic |
| 4 | 47834234 | 0.98 | rs7663935 | A | T | 0.72 | HRT | | | | 8 altered motifs | | | | CORIN | intronic |
| 4 | 47834743 | 0.93 | rs73238639 | A | G | 0.26 | HRT | | | | 18 altered motifs | | | | CORIN | intronic |
| 4 | 47837912 | 1 | rs2289433 | C | T | 0.72 | 22 tissues | 5 tissues | 6 tissues | | 6 altered motifs | | 5 hits | | CORIN | missense |
| SNP rs6823184 and variants with $r^2$> 0.8 | | | | | | | | | | | | | | | | |
| 4 | 47820797 | 0.99 | rs10049713 | C | T | 0.63 | | | | | RXRA | | | 2 hits | *CORIN* | intronic |
| 4 | 47831278 | 0.97 | rs6823698 | C | T | 0.63 | HRT | 9 tissues | 4 tissues | | 5 altered motifs | | 1 hit | 2 hits | *CORIN* | intronic |
| 4 | 47831771 | 1 | rs1866689 | A | C | 0.63 | HRT | PLCNT, MUS | HRT | | RP58 | | 1 hit | 2 hits | *CORIN* | intronic |
| 4 | 47836996 | 1 | rs6823184 | C | T | 0.63 | 22 tissues | GI | 20 tissues | CTCF | 4 altered motifs | | | 2 hits | *CORIN* | intronic |
| SNP rs10517195 and variants with $r^2$> 0.8 | | | | | | | | | | | | | | | | |
| 4 | 47680157 | 1 | rs10517195 | A | G | 0.15 | | | | | HNF4,RXRA | | 1 hit | | *CORIN* | synonymous |
| 4 | 47680697 | 0.97 | rs13111339 | T | C | 0.15 | | | | | Ik-2,Smad3 | | 1 hit | | *CORIN* | intronic |
| 4 | 47680757 | 0.97 | rs201021853 | TTATC | T | 0.15 | | | | | 30 altered motifs | | | | *CORIN* | intronic |
| 4 | 47680782 | 0.97 | rs13131207 | C | T | 0.15 | | | | | 8 altered motifs | | | | *CORIN* | intronic |
| 4 | 47680980 | 0.97 | rs28667091 | T | G | 0.15 | | | | | Mef2,Pou1f1 | | | | *CORIN* | intronic |
| 4 | 47680988 | 0.97 | rs17463016 | G | A | 0.15 | | | | | GCNF,HNF4,RXRA | | | | *CORIN* | intronic |

| | | | | | | | | | | | | |
|---|---|---|---|---|---|---|---|---|---|---|---|---|
| 4 | 47681326 | 0.97 | rs13135995 | G | A | 0.15 | | | ERalpha-a | | *CORIN* | intronic |
| 4 | 47681412 | 0.97 | rs13118104 | T | C | 0.15 | | | | 1 hit | *CORIN* | intronic |
| 4 | 47681439 | 0.97 | rs13137804 | C | A | 0.15 | | | 4 altered motifs | 1 hit | *CORIN* | intronic |
| 4 | 47682222 | 0.97 | rs7675211 | T | G | 0.15 | | | Pou2f2 | | *CORIN* | intronic |
| 4 | 47682271 | 0.96 | rs28879206 | A | C | 0.15 | | | Mef2,NF-kappaB | | *CORIN* | intronic |
| 4 | 47682996 | 0.92 | rs3792710 | A | G | 0.16 | | | GCM | | *CORIN* | intronic |
| 4 | 47683533 | 0.93 | rs12509695 | A | T | 0.15 | | | 8 altered motifs | | *CORIN* | intronic |
| 4 | 47684284 | 0.95 | rs3792709 | T | C | 0.15 | | | 13 altered motifs | | *CORIN* | intronic |
| 4 | 47684905 | 0.93 | rs13135597 | A | G | 0.15 | | | 16 altered motifs | | *CORIN* | intronic |
| 4 | 47685175 | 0.95 | rs7665841 | C | T | 0.15 | | | Foxp1 | | *CORIN* | intronic |
| 4 | 47686047 | 0.95 | rs7661217 | A | G | 0.15 | | BRN | 4 altered motifs | 1 hit | *CORIN* | intronic |
| 4 | 47686152 | 0.95 | rs7661414 | A | T | 0.15 | | | 12 altered motifs | | *CORIN* | intronic |
| 4 | 47686796 | 0.95 | rs4441728 | C | T | 0.15 | LIV | | Pou6f1,Sox | 1 hit | *CORIN* | intronic |
| 4 | 47686852 | 0.95 | rs4558839 | A | G | 0.15 | LIV | | | | *CORIN* | intronic |
| 4 | 47687581 | 0.95 | rs4271983 | T | C | 0.15 | LIV | | Mef2,Sox | | *CORIN* | intronic |
| 4 | 47687860 | 0.95 | rs4271984 | T | G | 0.15 | LIV | | GATA | 2 hits | *CORIN* | intronic |
| 4 | 47688040 | 0.95 | rs4273435 | T | C | 0.15 | LIV | | | | *CORIN* | intronic |
| 4 | 47689018 | 0.83 | rs10028293 | T | C | 0.15 | | | Mef2,NRSF,Pou3f2 | 1 hit | *CORIN* | intronic |
| SNP rs2271037 and variants with $r^2$> 0.8 | | | | | | | | | | | | |
| 4 | 47669973 | 0.87 | rs35736575 | T | C | 0.6 | MUS | MUS | Ets,TATA | | *CORIN* | intronic |
| 4 | 47672133 | 0.93 | rs12643755 | A | G | 0.59 | | | GR,RBP-Jkappa | 1 hit | *CORIN* | intronic |
| 4 | 47676842 | 0.99 | rs17601341 | G | A | 0.6 | | | 7 altered motifs | | *CORIN* | intronic |
| 4 | 47677911 | 0.98 | rs2271036 | T | C | 0.6 | | | GR,Hsf | | *CORIN* | intronic |
| 4 | 47678068 | 1 | rs2271037 | T | G | 0.6 | | | GR,Sox | | *CORIN* | intronic |
| SNP rs12509275 and variants with $r^2$> 0.8 | | | | | | | | | | | | |
| 4 | 47623205 | 1 | rs12332061 | G | A | 0.14 | MUS | | E2A | | *CORIN* | intronic |

| | | | | | | | | | | | | | | |
|---|---|---|---|---|---|---|---|---|---|---|---|---|---|---|
| 4 | 47623384 | 1 | rs7682364 | T | A | 0.14 | | STRM, MUS | | | NF-Y,STAT | | *CORIN* | intronic |
| 4 | 47623393 | 1 | rs7699738 | C | T | 0.14 | | STRM, MUS | | | THAP1 | 1 hit | *CORIN* | intronic |
| 4 | 47623794 | 1 | rs3215139 | TA | T | 0.14 | | STRM, BRST, MUS | IPSC,MUS,BRN | CTCF | CEBPB,Tgif1 | | *CORIN* | intronic |
| 4 | 47624017 | 1 | rs1344122 | C | T | 0.14 | | STRM, BRST, MUS | 5 tissues | P300 | | | *CORIN* | intronic |
| 4 | 47624315 | 1 | rs2292105 | C | G,T | 0.14 | | STRM, MUS, BRST | 4 tissues | | | | *CORIN* | intronic |
| 4 | 47624321 | 1 | rs7658542 | G | T | 0.14 | | STRM, MUS, BRST | 4 tissues | | EBF,Nkx2 | | *CORIN* | intronic |
| 4 | 47624344 | 1 | rs7658554 | G | A | 0.14 | | STRM, MUS, BRST | 4 tissues | | Mef2,Pax-5 | | *CORIN* | intronic |
| 4 | 47624399 | 1 | rs7658596 | G | A | 0.14 | | STRM, MUS | SKIN,MUS | | 4 altered motifs | | *CORIN* | intronic |
| 4 | 47624569 | 1 | rs7660319 | C | T | 0.14 | | STRM, MUS | | | Foxp3,Sox | | *CORIN* | intronic |
| 4 | 47624832 | 1 | rs7660901 | C | T | 0.14 | | STRM, MUS | | | 5 altered motifs | | *CORIN* | intronic |
| 4 | 47624892 | 1 | rs13107754 | G | C | 0.14 | | STRM, MUS | | | 7 altered motifs | | *CORIN* | intronic |
| 4 | 47625109 | 1 | rs9291309 | C | T | 0.14 | | STRM | | | CEBPB | | *CORIN* | intronic |
| 4 | 47625186 | 1 | rs9291310 | A | G | 0.14 | | STRM | | | 7 altered motifs | | *CORIN* | intronic |
| 4 | 47625424 | 1 | rs9283674 | A | G | 0.14 | | STRM | | | 20 altered motifs | | *CORIN* | intronic |
| 4 | 47625909 | 1 | rs12508056 | C | T | 0.14 | | | | | 4 altered motifs | | *CORIN* | intronic |
| 4 | 47626135 | 1 | rs11941312 | T | G | 0.14 | | | | | 7 altered motifs | | *CORIN* | intronic |
| 4 | 47626684 | 1 | rs10005710 | C | T | 0.14 | | | BRN | | FXR,RXRA | | *CORIN* | intronic |
| 4 | 47626971 | 0.86 | rs111543039 | G | GGTT | 0.15 | | | | | 6 altered motifs | | *CORIN* | intronic |
| 4 | 47627034 | 1 | rs10029881 | G | A | 0.14 | | | | | HP1-site-factor | | *CORIN* | intronic |
| 4 | 47627066 | 1 | rs10029890 | G | C | 0.14 | | | | | ERalpha-a,Smad3 | | *CORIN* | intronic |
| 4 | 47627623 | 1 | rs12504395 | C | T | 0.14 | | | | | Hand1,Irf,Spdef | | *CORIN* | intronic |

| | | | | | | | | | | |
|---|---|---|---|---|---|---|---|---|---|---|
| 4 | 47627753 | 1 | rs13142266 | G | C | 0.14 | | | *CORIN* | intronic |
| 4 | 47627849 | 1 | rs12509275 | T | G | 0.14 | Gfi1b | 1 hit | *CORIN* | intronic |
| 4 | 47628033 | 1 | rs4586905 | G | A | 0.14 | SPIB,STAT,p300 | | *CORIN* | intronic |
| 4 | 47628044 | 1 | rs4499673 | C | T | 0.14 | AP-3 | | *CORIN* | intronic |
| 4 | 47628293 | 1 | rs4522838 | T | G | 0.14 | 5 altered motifs | | *CORIN* | intronic |
| 4 | 47628461 | 1 | rs4388056 | T | C | 0.14 | Egr-1,Foxp1,RREB-1 | | *CORIN* | intronic |
| 4 | 47628564 | 1 | rs13149126 | G | A | 0.14 | GR | | *CORIN* | intronic |
| 4 | 47628588 | 1 | rs35971161 | A | G | 0.14 | 4 altered motifs | | *CORIN* | intronic |
| 4 | 47628606 | 1 | rs34131087 | A | G | 0.14 | 5 altered motifs | | *CORIN* | intronic |
| 4 | 47628837 | 1 | rs10023732 | A | G | 0.14 | HMG-IY,SIX5 | | *CORIN* | intronic |
| 4 | 47629188 | 1 | rs4695257 | T | A | 0.14 | 5 altered motifs | | *CORIN* | intronic |
| 4 | 47629503 | 0.99 | rs4695258 | C | T | 0.14 | 4 altered motifs | | *CORIN* | intronic |
| 4 | 47629566 | 1 | rs4695259 | T | C | 0.14 | 6 altered motifs | | *CORIN* | intronic |
| 4 | 47629966 | 1 | rs13105718 | G | A | 0.14 | CEBPG | | *CORIN* | intronic |
| 4 | 47630138 | 1 | rs4695260 | G | A | 0.14 | 10 altered motifs | | *CORIN* | intronic |
| 4 | 47630419 | 1 | rs4695261 | G | A | 0.14 | 4 altered motifs | | *CORIN* | intronic |
| 4 | 47630627 | 1 | rs12512399 | T | C | 0.14 | TATA | | *CORIN* | intronic |
| 4 | 47631027 | 1 | rs1875823 | C | T | 0.14 | 5 altered motifs | | *CORIN* | intronic |
| 4 | 47631039 | 1 | rs1875824 | A | C | 0.14 | Gfi1,KAP1 | | *CORIN* | intronic |
| 4 | 47632247 | 1 | rs28549266 | C | T | 0.14 | 4 altered motifs | | *CORIN* | intronic |
| 4 | 47632405 | 1 | rs16860493 | A | G | 0.14 | Foxp3,Hdx | | *CORIN* | intronic |
| 4 | 47632811 | 0.94 | rs57700234 | A | G | 0.13 | | | *CORIN* | intronic |
| 4 | 47632969 | 1 | rs13127813 | G | A | 0.14 | 4 altered motifs | | *CORIN* | intronic |
| 4 | 47632997 | 1 | rs13127836 | G | A | 0.14 | BDP1 | | *CORIN* | intronic |
| 4 | 47633035 | 1 | rs13133141 | G | A | 0.14 | 6 altered motifs | | *CORIN* | intronic |
| 4 | 47633113 | 1 | rs4694862 | T | C | 0.14 | 4 altered motifs | | *CORIN* | intronic |

| | | | | | | | | | | | | | |
|---|---|---|---|---|---|---|---|---|---|---|---|---|---|
| 4 | 47633466 | 1 | rs4695262 | T | C | 0.14 | | | | Foxo,Hbp1,Pou5f1 | | *CORIN* | intronic |
| 4 | 47633569 | 0.97 | rs4694863 | A | C | 0.14 | | | | Ets,GATA,RP58 | 6 hits | *CORIN* | intronic |
| 4 | 47633995 | 0.99 | rs9918055 | C | A | 0.14 | | | | 13 altered motifs | | *CORIN* | intronic |
| 4 | 47634033 | 1 | rs9918069 | C | T | 0.14 | | | | 7 altered motifs | | *CORIN* | intronic |
| 4 | 47634049 | 1 | rs9918070 | C | T | 0.14 | | | | Mef2 | | *CORIN* | intronic |
| 4 | 47634097 | 0.96 | rs9917894 | T | C | 0.13 | | | | 10 altered motifs | | *CORIN* | intronic |
| 4 | 47634136 | 1 | rs9917878 | A | T | 0.14 | | | | 6 altered motifs | | *CORIN* | intronic |
| 4 | 47634444 | 0.96 | rs11932585 | A | G | 0.13 | | | | GR | | *CORIN* | intronic |
| 4 | 47634454 | 0.96 | rs11933636 | T | C | 0.13 | | | | | | *CORIN* | intronic |
| 4 | 47634542 | 1 | rs7689937 | G | C | 0.14 | | | | AP-1,Isl2,NF-E2 | | *CORIN* | intronic |
| 4 | 47635112 | 1 | rs7679354 | T | C | 0.14 | | | | CTCF,GATA | | *CORIN* | intronic |
| 4 | 47635274 | 1 | rs35932402 | A | G | 0.14 | | | | | | *CORIN* | intronic |
| 4 | 47635406 | 1 | rs17462720 | T | G | 0.14 | | | | BDP1 | | *CORIN* | intronic |
| 4 | 47636308 | 1 | rs28706404 | C | T | 0.14 | | | | 5 altered motifs | | *CORIN* | intronic |
| 4 | 47637296 | 1 | rs117041780 | G | C | 0.14 | | | | 5 altered motifs | | *CORIN* | intronic |
| 4 | 47637349 | 1 | rs28848023 | G | A | 0.14 | | | | GATA,TATA | | *CORIN* | intronic |
| 4 | 47637662 | 0.99 | rs11933930 | G | A | 0.14 | | | | Nkx2 | | *CORIN* | intronic |
| 4 | 47637680 | 0.97 | rs11943208 | C | A | 0.14 | | | | Ik-1 | | *CORIN* | intronic |
| 4 | 47637699 | 0.97 | rs11947985 | A | G | 0.14 | | | | Ets,p53 | | *CORIN* | intronic |
| 4 | 47638445 | 1 | rs7665081 | T | C | 0.14 | | | | 4 altered motifs | | *CORIN* | intronic |
| 4 | 47639080 | 1 | rs1317157 | C | G | 0.14 | | | | Maf,Pax-4 | | *CORIN* | intronic |
| 4 | 47639144 | 1 | rs1317158 | G | A | 0.14 | | | | Hand1 | | *CORIN* | intronic |
| 4 | 47639198 | 1 | rs1317159 | G | A | 0.14 | | | | GR,Hbp1 | | *CORIN* | intronic |
| 4 | 47639700 | 1 | rs4695263 | C | G | 0.14 | 11 tissues | 12 tissues | TCF4 | 11 altered motifs | | *CORIN* | intronic |
| 4 | 47639778 | 1 | rs4695264 | G | A | 0.14 | 11 tissues | 12 tissues | TCF4 | 6 altered motifs | | *CORIN* | intronic |
| 4 | 47640000 | 0.84 | rs202115654 | T | TC | 0.12 | 4 tissues | | | 5 altered motifs | | *CORIN* | intronic |

| | | | | | | | | | | | |
|---|---|---|---|---|---|---|---|---|---|---|---|
| 4 | 47640032 | 1 | rs10938497 | G | T | 0.14 | | | NF-AT1 | *CORIN* | intronic |
| 4 | 47640335 | 0.96 | rs12331236 | T | C | 0.13 | FAT, MUS, SKIN | | Zfp187 | *CORIN* | intronic |
| 4 | 47640520 | 1 | rs10213618 | C | T | 0.14 | | | 5 altered motifs | *CORIN* | intronic |
| 4 | 47640756 | 0.97 | rs10213076 | G | A | 0.14 | | | AhR,LRH1,Zec | *CORIN* | intronic |
| 4 | 47640815 | 1 | rs10212843 | A | G | 0.14 | | | GATA,Hltf,Lmo2-complex | *CORIN* | intronic |
| 4 | 47640989 | 1 | rs10212847 | A | G | 0.14 | | | 5 altered motifs | *CORIN* | intronic |
| 4 | 47641008 | 1 | rs9291312 | G | A | 0.14 | 5 tissues | | GZF1 | *CORIN* | intronic |
| 4 | 47641033 | 1 | rs10212955 | T | C | 0.14 | | SKIN | | *CORIN* | intronic |
| 4 | 47641050 | 1 | rs9291313 | G | T | 0.14 | 5 tissues | SKIN,SKIN,SKIN | AIRE,STAT | *CORIN* | intronic |
| 4 | 47641069 | 1 | rs10213124 | G | T | 0.14 | | 4 tissues | GR,YY1 | *CORIN* | intronic |
| 4 | 47641078 | 1 | rs9291314 | G | A | 0.14 | 5 tissues | 5 tissues | Pou2f2 | *CORIN* | intronic |
| 4 | 47641162 | 1 | rs10005066 | T | G | 0.14 | | 5 tissues | 6 altered motifs | *CORIN* | intronic |
| 4 | 47641424 | 1 | rs10013225 | G | C | 0.14 | | | | *CORIN* | intronic |
| 4 | 47641560 | 0.99 | rs3817091 | T | A | 0.14 | 5 tissues | | 4 altered motifs | *CORIN* | intronic |
| 4 | 47649428 | 0.97 | rs1317606 | G | A | 0.14 | | | Smad3,VDR | *CORIN* | intronic |
| 4 | 47658445 | 0.93 | rs28793954 | T | C | 0.15 | | | 4 altered motifs | *CORIN* | intronic |
| 4 | 47658767 | 0.93 | rs1578977 | T | C | 0.15 | | | HLF,Pou2f2,Pou5f1 | *CORIN* | intronic |
| 4 | 47659474 | 0.93 | rs1573804 | C | G | 0.15 | | | 4 altered motifs | *CORIN* | intronic |
| 4 | 47659781 | 0.92 | rs10029017 | T | C | 0.15 | | | Pou2f2 | *CORIN* | intronic |
| 4 | 47661029 | 0.93 | rs13146115 | A | G | 0.15 | | | Ik-2 | *CORIN* | intronic |
| 4 | 47662640 | 0.92 | rs3792712 | A | G | 0.15 | | | 4 altered motifs | *CORIN* | intronic |
