# Supplementary material for: Associations between potentially functional CORIN SNPs and serum corin levels in the Chinese Han population
Source: BMC Genet. 2019 Dec 19;20:99. doi: 10.1186/s12863-019-0802-4 (PMC6923953; doi:10.1186/s12863-019-0802-4)
Supplement: Supplementary file 3 — Additional file 3: Table S3. Significant associations between CORIN SNPs and methylations. The results showed 12 significant associations between 12 SNPs and cg02955940 methylation sites in CORIN gene (FDR < 0.05). [file 12863_2019_802_MOESM3_ESM.pdf]

Supplementary Table S3 Significant associations between *CORIN* SNPs and methylations

SNPs	SNP position†	Gene	Methylation position†	Statistic	*P* value	FDR	beta	se
rs10049713	47822814	cg02955940	47841314	6.41	1.38E-07	0.00050	0.0449	0.0070
rs6823698	47833295	cg02955940	47841314	6.38	1.54E-07	0.00050	0.0446	0.0070
rs1866689	47833788	cg02955940	47841314	6.38	1.54E-07	0.00050	0.0446	0.0070
rs6823184	47839013	cg02955940	47841314	6.38	1.54E-07	0.00050	0.0446	0.0070
rs1440227	47832892	cg02955940	47841314	5.31	4.65E-06	0.00731	0.0493	0.0093
rs7663935	47836251	cg02955940	47841314	5.31	4.65E-06	0.00731	0.0493	0.0093
rs2289433	47839929	cg02955940	47841314	5.31	4.76E-06	0.00731	0.0492	0.0093
rs4695277	47823057	cg02955940	47841314	-5.17	7.39E-06	0.00731	-0.0506	0.0098
rs73238638	47825120	cg02955940	47841314	-5.17	7.39E-06	0.00731	-0.0506	0.0098
rs4694867	47827928	cg02955940	47841314	-5.17	7.39E-06	0.00731	-0.0506	0.0098
rs1583808	47836096	cg02955940	47841314	-5.17	7.39E-06	0.00731	-0.0506	0.0098
rs73238639	47836760	cg02955940	47841314	-5.17	7.39E-06	0.00731	-0.0506	0.0098

†: Assembly: GRCh37.

The four SNPs in red are in strong LD. Their physical locations were shown in Figure 2. rs6823184 is the tagSNP in this study.

The eight SNPs in blue are in strong LD. Their physical locations were shown in Figure 2. rs2289433 is the tagSNP in this study.
