# Supplementary material for: Associations between potentially functional CORIN SNPs and serum corin levels in the Chinese Han population
Source: BMC Genet. 2019 Dec 19;20:99. doi: 10.1186/s12863-019-0802-4 (PMC6923953; doi:10.1186/s12863-019-0802-4)
Supplement: Supplementary file 4 — Additional file 4: Table S4. Summary association data for GSMR analysis. This table contained the formatted summary-level data that was used in the GSMR analysis. [file 12863_2019_802_MOESM4_ESM.pdf]

Supplementary Table S4 Summary association data for gsmr analysis

SNP	a1	a2	a1_freq	bzx	bzx_se	bzx_pval	bzx_n	bzy	bzy_se	bzy_pval	bzy_n
rs2289433	G	A	0.343	0.0492	0.0093	4.76E-06	43	0.037	0.0180	0.0399	1462
rs6823184	C	T	0.435	0.0446	0.0070	1.54E-07	43	0.038	0.0169	0.0238	1459
rs10008014	C	T	0.246	0.0407	0.0118	1.31E-03	43	0.000	0.0199	0.9904	1462
rs17654423	C	T	0.266	0.0343	0.0106	2.53E-03	43	-0.019	0.0188	0.3191	1462
rs10517195	G	A	0.157	0.0141	0.0164	3.94E-01	43	0.074	0.0233	0.0016	1462
rs2271037	T	C	0.387	-0.0019	0.0124	8.79E-01	43	0.042	0.0174	0.0148	1462
rs2351784	T	C	0.230	-0.0135	0.0147	3.63E-01	43	-0.012	0.0205	0.5648	1462
rs12509275	G	T	0.138	0.0334	0.0188	8.29E-02	43	0.070	0.0242	0.0038	1446
rs3749585	C	T	0.480	-0.0093	0.0106	3.89E-01	43	-0.006	0.0168	0.7367	1462

a1: minor allele

a2: major allele

a1_freq: frequency of a1

bzx: the effect size of a1 on cg02955940 methylation level

bzx_se: standard error of bzx

bzx_pval: p value for bzx

bzx_n: per-SNP sample size of SNP-methylation association

bzy: the effect size of a1 on corin level

bzy_se: standard error of bzy

bzy_pval: p value for bzy

bzy_n: per-SNP sample size of SNP-corin association
